# Literacy and health outcomes: a cross-sectional study in 1002 adults with diabetes

**DOI:** 10.1186/1471-2296-7-49

**Published:** 2006-08-14

**Authors:** Nancy S Morris, Charles D MacLean, Benjamin Littenberg

**Affiliations:** 1 College of Nursing and Health Sciences, University of Vermont, Burlington, Vermont, USA; 2 College of Medicine, University of Vermont, Burlington, Vermont, USA

## Abstract

**Background:**

Inconsistent findings reported in the literature contribute to the lack of complete understanding of the association of literacy with health outcomes. We evaluated the association between literacy, physiologic control and diabetes complications among adults with diabetes.

**Methods:**

A cross-sectional study of 1,002 English speaking adults with diabetes, randomly selected from the Vermont Diabetes Information System, a cluster-randomized trial of a diabetes decision support system in a region-wide sample of primary care practices was conducted between July 2003 and March 2005. Literacy was assessed by the Short-Test of Functional Health Literacy in Adults. Outcome measures included glycated hemoglobin, low density lipoprotein, blood pressure and self-reported complications.

**Results:**

After adjusting for sociodemographic characteristics, duration of diabetes, diabetes education, depression, alcohol use, and medication use we did not find a significant association between literacy and glycemic control (beta coefficent,+ 0.001; 95% confidence interval [CI], -0.01 to +0.01; *P *= .88), systolic blood pressure (beta coefficent, +0.08; 95% CI, -0.10 to +0.26; *P *= .39), diastolic blood pressure (beta coefficent, -0.03; 95% CI, -0.12 to +0.07, *P *= .59), or low density lipoprotein (beta coefficent, +0.04; 95% CI, -0.27 to +0.36, *P *= .77. We found no association between literacy and report of diabetes complications.

**Conclusion:**

These findings suggest that literacy, as measured by the S-TOFHLA, is not associated with glycated hemoglobin, blood pressure, lipid levels or self-reported diabetes complications in a cross-sectional study of older adults with diabetes under relatively good glycemic control. Additional studies to examine the optimal measurement of health literacy and its relationship to health outcomes over time are needed.

## Background

Navigating the US health care system requires sophisticated reading, writing, and numeracy skills that go beyond the health literacy abilities of many Americans [1,2]. Adults with limited health literacy face challenges interpreting and analyzing health information, becoming knowledgeable about their specific health conditions and health risks, and understanding prescriptions and other treatment recommendations [3-9]. Evidence is beginning to accumulate about the prevalence of limited health literacy and its association with the use of health care services [5-7,10-13] and health outcomes[8,14-16].

Patients with chronic conditions such as diabetes face even greater communication challenges. The complexity of diabetes care requires an informed individual who can seek, obtain, and comprehend information to engage in the management of his/her health. Health outcomes for adults with diabetes are better for those who can optimally incorporate self-management of their diabetes into their daily lives[17]. Decreasing elevated BP, LDL, and A1C in patients with type 2 diabetes reduces the risk of cardiovascular and microvascular events by about 50 percent[18]. The American Diabetes Association recommends that best management of diabetes can be obtained with a glycated hemoglobin (A1C) < 7%, LDL < 100 mg/dl, and BP < 130/80 mmHg[19].

Recent cross-sectional studies have assessed the association between literacy and diabetes care with conflicting outcomes. Williams *et al*.[9] did not find a significant correlation between literacy and health outcomes while Schillinger *et al*.[16] reported worse glycemic control and higher rates of retinopathy and cerebrovascular complications in subjects with lower literacy. Ross *et al*.[20] did not find a significant correlation between the literacy of children and their glycemic control but they did observe a significant relationship between the literacy of the mother and her child's glycemic control. Two recent studies by Rothman and colleagues[21,22] examined the relationship between literacy and achievement of goal A1C specifically in disease management programs and reported greater benefit to patients with I literacy.

Given the complexity of diabetes management, we hypothesized that adults with diabetes and limited literacy would be less likely to achieve the recommended goals for A1C, systolic blood pressure (SBP), diastolic blood pressure (DBP), and low density lipoprotein (LDL). We also hypothesized that adults with diabetes and limited literacy would report more complications related to their diabetes.

## Methods

### Setting and study participants

This study was part of the Vermont Diabetes Information System (VDIS), a cluster-randomized trial of a diabetes decision support system in a region-wide sample of primary care practices. Characteristics of the VDIS registry, study design and data-collection procedures have been described[23]. A field survey targeted at a sub-sample of subjects with confirmed diabetes was designed to provide a better understanding of variables associated with health outcomes. VDIS subject names were randomly sorted and patients contacted by telephone until a sample of approximately 15% of the patients from each practice agreed to participate in an interview. Demographic information including sex, race, ethnicity, education, income, and marital status as well as data on smoking, drinking and exercise habits; co-morbidity; diabetes self care activities; duration of diabetes; and depressive symptoms were obtained by questionnaire which was mailed to subjects prior to an in-home interview. All of the written survey materials were written at a mean Flesch-Kincaid Grade level of 6.2. Except for completion of the S-TOFHLA, subjects received help in reading and interpreting survey questions if they requested assistance.

At the time of the interview, a research assistant reviewed the questionnaire for completeness and asked the subjects to complete overlooked items, recorded medication use by checking actual medication containers in the home, measured height, weight, and blood pressure, and administered the Short Test of Functional Health Literacy for Adults (S-TOFHLA)[24] and the Patient Health Questionnaire-9[25]. Interviews occurred between July 2003 and March 2005. The University of Vermont Committee on Human Research in the Medical Sciences approved the protocol for this study and all subjects gave written informed consent to participate.

Our study power was adequate to detect an important association between literacy and physiologic control. Given the number of subjects recruited and the observed standard deviations, we had 84% power for detecting a difference in A1C levels of 0.3% between subjects with limited versus adequate literacy. Similarly, we had 79% power to detect a 5 mmHg difference in blood pressure. For LDL, we had 83% power for a difference of 8 mg/dl.

### Measures

The S-TOFHLA, a 36-item, 7-minute, timed test of reading comprehension, employs the Cloze procedure, in which a word in a sentence is omitted and must be chosen from a multiple choice list. It uses passages from instructions for preparation for an upper gastrointestinal series and a section of a Medicaid application. Results are categorized into inadequate (0–16 correct answers), marginal (17–22), and adequate health literacy (23–36). The S-TOFHLA has good internal consistency (Cronbach's alpha = 0.98 for all items combined) and concurrent validity compared to the long version of the TOFHLA (r = 0.91)[24].

Selected variables (Table 1) from the field survey associated with glycemic, lipid, or blood pressure control were used for this analysis. Subjects self-reported their sex, income, education, marital status, race/ethnicity, health insurance, tobacco and alcohol use, co-morbidities, and diabetes self care activities. Race and ethnicity were included because of their documented relationship to health literacy[2,22].

**Table 1:** Baseline Characteristics of 1,002 Adults with Diabetes by Literacy Level

*Characteristics*	*All Subjects*	*Inadequate Literacy*	*Marginal Literacy*	*Adequate Literacy*	*P Value*†
Number of subjects (%)	1002 (100)	105 (10)	66 (7)	831 (83)	
					
STOFHLA Score, range	0–36	0–16	17–22	18–36	
S-TOFHLA Score, median (IQR)	34 (29–35)	0 (0–12)	20 (18–21)	35 (33–35)	<0.001
					
Age, median (IQR), y	66 (57–74)	74 (67–79)	74 (67–79)	64 (56–72)	<0.001
Female, No. (%)	545 (54)	51 (49)	34 (52)	460 (55)	0.37
White race, No. (%)	972 (97)	97 (94)	65 (98)	810 (98)	0.11
Married or living as married, No. (%)	626 (63)	50 (48)	41 (62)	535 (65)	0.005
Annual income < $30,000, No. (%)	545 (59)	85 (92)	43 (75)	417 (54)	<0.001
Education, No. (%)					
Some high school or less	245 (25)	72 (69)	31 (48)	142 (17)	<0.001
High school graduate	354 (36)	24 (23)	19 (30)	311 (38)	
College graduate/some college	305 (31)	6 (6)	11 (17)	288 (35)	
Graduate education	91 (9)	2 (2)	3 (5)	86 (10)	
Insurance, No. (%)*					
Private insurance	582 (58)	37 (36)	33 (51)	512 (62)	<0.001
Medicare insurance	594 (60)	91 (88)	58 (89)	445 (54)	<0.001
Medicaid insurance	212 (21)	48 (47)	14 (22)	150 (18)	<0.001
Military or VA insurance	51 (5)	3 (3)	6 (9)	42 (5)	0.19
No insurance	24 (2)	0 (0)	2 (3)	22 (3)	0.19
					
Alcohol intake >1dk/wk, No. (%)	194 (20)	10 (10)	8 (12)	176 (22)	0.003
					
Years with diabetes, median (IQR)	6.8 (3–14)	9.5 (4–20)	10.5 (4–20)	6.3 (3–13)	0.01
Attended diabetes class, No. (%)	349 (35)	26 (25)	21 (32)	302 (37)	0.06
					
Treatments for diabetes, No. (%)					
Diet alone	242 (24)	20 (19)	7 (11)	215 (26)	< 0.001
Oral hypoglycemic alone	574 (57)	64 (61)	44 (67)	466 (56)	
Insulin alone	93 (9)	19 (18)	5 (8)	69 (8)	
Insulin and oral agent	92 (9)	2 (2)	10 (15)	80 (10)	
					
Hypertension medication, No. (%)	834 (83)	91 (87)	61 (92)	682 (82)	0.06
Cholesterol medication, No. (%)	591 (59)	57 (54)	43 (65)	491 (59)	0.36
					
A1C, median (IQR)	6.9 (6.3–7.7)	6.9 (6.3–7.7)	6.8 (6.3–7.3)	6.9 (6.3–7.7)	0.50
Systolic Blood Pressure, median (IQR)	139 (127–151)	137 (123–159)	144 (131–155)	138 (127–150)	0.17
Diastolic Blood Pressure, median (IQR)	79 (71–85)	76 (68–83)	77 (68–84)	79 (72–86)	0.003
LDL-cholesterol, median (IQR)	99 (83–118)	99 (79–117)	94 (74–106)	99 (84–119)	0.06
					
Complications, No. (%)					
Retinopathy	189(20)	29 (30)	21 (34)	139 (18)	<0.001
Nephropathy	44 (9)	8 (15)	0 (0)	36 (9)	0.11
Gastroparesis	56 (6)	9 (9)	6 (10)	41 (6)	0.16
Foot/leg problems	288 (31)	30 (30)	27 (44)	231 (30)	0.07
Cerebrovascular disease	118 (12)	22 (21)	11 (17)	85 (10)	0.003
Coronary artery disease	194 (19)	32 (30)	18 (27)	144 (17)	0.002
					
Depression; PHQ > 5, No. (%); N = 589	195 (33)	24 (40)	14 (54)	157 (31)	0.03
Depression score (0–27), median (IQR)	2 (0–6)	3 (1–8)	5 (2–7)	2 (0–6)	0.04

*Many subjects had more than one health insurance type.

†Fisher's Exact test was used for categorical variables; The Kruskal-Wallis test, adjusted for ties, was used for continuous variables.

Abbreviations: Glycated hemoglobin (A1C); Interquartile range (IQR); Low Density Lipoprotein (LDL); Number (No); Patient Health Questionnaire (PHQ).

Because of the association between depression and glycemic control [26-28], we measured depressive symptoms with the Patient Health Questionnaire-9 (PHQ-9). This is a brief self-report instrument that quantifies the presence and severity of depression[25]. The PHQ-9 scores the self-reported frequency of each of the nine Diagnostic and Statistical Manual, 4^th ^edition depression criteria on a scale of 0 (not at all) to 3 (nearly every day). A score of 10 or more has been documented to have a sensitivity of 88% and a specificity of 88% for major depression[25].

We obtained data about kidney disease, heart disease, and cerebrovascular disease from the Self-Administered Comorbidity Questionnaire, a modification of the Charlson Index that has moderately strong associations with a standard medical record-based co-morbidity measure[29]. To identify complications of diabetes, we asked patients whether their doctor or health care provider had ever told them that they had "problems with vision" (retinopathy), "pain, burning, or numbness in the feet or legs" (neuropathy), "problems with stomach emptying" (gastroparesis), or "ulcers or sores on leg or foot" related to their diabetes.

The research assistant measured height using a portable stadiometer (SECA, Inc.), weight with a portable scale (Healthometer model HAP200KD-41), and blood pressure with an automated sphygmomanometer (Omron model HEM-711). Blood pressure was obtained in the seated position in the left arm (unless contraindicated) using the cuff size recommended by the manufacturer. Three readings were obtained at five-minute intervals and averaged for the final result. We obtained each patient's most recent A1C and cholesterol levels directly from their local clinical laboratories[23].

### Statistical analysis

The primary outcomes (A1C, SBP, DBP, and LDL) were analyzed as continuous variables. We analyzed literacy as both a continuous variable and a categorical variable: inadequate literacy (S-TOFHLA score 0–16), marginal literacy (S-TOFHLA score 17–22) and adequate literacy (S-TOFHA score 23–36).

Regression analysis was used to measure the association between the S-TOFHLA score and each of our four outcomes (A1C, SBP, DBP, and LDL) after controlling for potentially confounding patient characteristics. We performed multivariate linear regression for each primary outcome controlling for variables shown to be important clinically or in other studies[16,22]. Specifically, we controlled for age, sex, race/ethnicity, marital status, insurance, depression, alcohol use, diabetes education, duration of diabetes, and as appropriate, medication use specific to the management of blood pressure, serum glucose, or cholesterol.

Subjects with major cognitive impairment were excluded from the VDIS trial;[23] nonetheless, we repeated the analyses after excluding subjects with self-reported stroke, Alzheimer disease or dementia, to eliminate the possibility that a low S-TOFHLA score may result from potentially significant cognitive problems. We also repeated the analyses after excluding subjects with limited literacy due to poor vision or other physical impairment as is often done in studies assessing health literacy.

We determined the effects of literacy on self-reported retinopathy, neuropathy, gastroparesis, foot and leg ulcerations, cerebrovascular disease, and coronary artery disease, all common complications of diabetes. We performed multivariate logistic regression for each complication, controlling for age, sex, race/ethnicity, marital status, insurance, income, depression, alcohol use, diabetes education, duration of diabetes, hypertension, and medication use specific to the management of blood pressure, serum glucose, or cholesterol. We also accounted for clustering of patients with physicians. For coronary heart disease, cerebrovascular disease, and foot and leg problems related to diabetes, we also adjusted for smoking. All analyses were performed with STATA 8.2 (StataCorp, College Station, Texas).

## Results

Of the 1,576 patients we attempted to contact to participate in the interview, 36% (570) were not reached or declined. The 7,801 non-interviewed subjects of VDIS, (which includes those who declined and those never contacted) were younger (mean age of 63 versus 65, *P *< 0.001); more likely to be men (49% versus 46%; *P *= 0.06); had a lower baseline A1C (mean of 6.9 versus 7.1, *P *< 0.001); and a higher baseline LDL (mean of 106 versus 102, *P *= 0.002) than those who completed the interview. Due to missing data on 4 subjects, we had complete questionnaire results and S-TOFHLA scores on 1002 subjects with confirmed diabetes. The demographic characteristics of the study population reflect the population of northern New England (Table 1)[30]. The subjects were predominately white, educated, and older with a mean age of almost 65 years. Most had health insurance, were diagnosed with diabetes on average 7 years and almost half had an A1C < 7%. Overall, the mean A1C was 7.12%, the mean LDL was 102 mg/dl, and the mean BP was 140/78 mmHg. The mean S-TOFHLA score was 29.7 with a range of 0–36.

One-hundred and five (10%) subjects had inadequate literacy, 66 (7%) had marginal literacy, and 831 (83%) had adequate literacy (Table 1). Compared to those with adequate literacy, subjects with inadequate or marginal literacy were significantly older; less educated; less likely to be married; poorer; less likely to have private insurance and more likely to have Medicare or Medicaid coverage. Those with lower literacy had been diagnosed with diabetes for a longer time; reported less alcohol use; were more likely to have depression; had a lower diastolic blood pressure; more self-reported retinopathy, stroke or coronary artery disease; and were more likely to take medication for diabetes and hypertension.

In unadjusted linear regression analyses there were no significant relationships for any of the four outcome variables except diastolic blood pressure. However, after adjusting for age, sex, race, marital status, insurance, income, duration of diabetes, diabetes education, depression, alcohol use, and medication use specific to each outcome, literacy was no longer independently associated with any of the outcomes of interest (Table 2).

**Table 2:** Relationship between Literacy* and Physiologic Control in 1,002 Adults with Diabetes

*Physiologic parameter*	*Unadjusted*		*Adjusted*^†^	
		
	*Coefficient (95% CI)*	*P*	*Coefficient (95% CI)*	*P*
A1C (%/point)	+0.002 (-0.006, +0.010)	.65	-0.001 (-0.012, +0.014)	.88
LDL (mg/dl/point)	+0.196 (-0.018, +0.410)	.07	+0.445 (-0.267, +0.356)	.78
SBP(mmHg/point)	-0.060 (-0.187, +0.068)	.36	+ 0.079 (-0.103, +0.262)	.39
DBP(mmHg/point)	+ 0.140 (+0.073, +0.208)	<0.001	-0.026 (-0.118, +0.067)	.59

*STOFHLA score (assessment of literacy) examined as a continuous measure from 0–36; score was assigned 0 if the subject could not read due to poor vision or other impairment.

^†^Adjusted for age, sex, race, marital status, insurance, income, duration of diabetes, diabetes education, depression, alcohol use, and medication use specific to each outcome.

Repeating the analyses after excluding subjects who reported a history of stroke (N = 118) or dementia (N = 5) did not identify a significant relationship between S-TOFHLA score and A1C, LDL, or blood pressure. Likewise, excluding subjects with poor vision or other physical impairments (N = 23) revealed no significant associations between literacy and physiologic control of diabetes.

More complications related to diabetes were reported by the subjects with inadequate or marginal literacy. However, the differences between the groups disappeared after adjusting for confounders. (Table 3).

**Table 3:** Adjusted Odds of Self-reported Diabetes Complications for Subjects with Inadequate and Marginal Literacy Compared to Adequate Literacy

*Complication*	*No. of Study Subjects with Complication*.		Odds Ratio (95% CI)*	*P Value*
Retinopathy	Inadequate	29 (30%)	+ 1.88 (+0.90, +3.91)	.09
	Marginal	21 (34%)	+ 2.30 (+0.63, +8.44)	.21
	Adequate	139 (18%)	+ 1.0	
				
Nephropathy	Inadequate	8 (15%)	+1.05 (+0.39, +2.80)	.93
	Marginal	0 (0%)	+ 0.99 (+0.95, +1.03)	.53
	Adequate	36 (9%)	+ 1.0	
				
Foot/Leg Problems	Inadequate	30 (30%)	+0.52 (+0.24, +1.16)	.11
	Marginal	27 (44%)	+ 1.39 (+0.47, +4.12)	.55
	Adequate	231 (30%)	+ 1.0	
				
Gastroparesis	Inadequate	9 (9%)	+ 1.92 (+0.58, +6.36)	.28
	Marginal	6 (10%)	+ 1.98 (+0.26, +18.07)	.55
	Adequate	41 (6%)	+ 1.0	
				
Coronary artery disease	Inadequate	32 (30%)	+ 0.76 (+0.36, +1.63)	.49
	Marginal	18 (27%)	+ 1.12 (+0.34, +3.70)	.85
	Adequate	144 (17%)	+ 1.0	
				
Cerebrovascular disease	Inadequate	22 (21%)	+ 0.86 (+0.39, +1.91)	.72
	Marginal	11 (17%)	+ 0.65 (+1.66, +2.57)	.54
	Adequate	85 (10%)	+ 1.0	

*Adjusted for age, sex, race, marital status, insurance, income, duration of diabetes, diabetes education, depression, alcohol use, hypertension, and medication use specific for blood pressure, diabetes, or lipid management and accounting for clustering of patients with physicians. Smoking was included in the models for foot/leg conditions, cerebrovascular disease, and coronary artery disease.

## Discussion

After controlling for potential confounders, we did not find a significant association between literacy and health outcomes for glycemic control, blood pressure, or dyslipidemia in adults who receive diabetes care in community primary care settings. We also did not find an association between literacy and complications of diabetes. This is counter to our expectations, particularly for a study population with a chronic disease requiring self-care to achieve optimal health. Our results raise questions regarding how health literacy is assessed, how it may vary across populations, and how patients may compensate for low literacy.

Our results are consistent with those of Williams *et al*.[9] who reported no significant association between control of blood glucose and blood pressure and lower literacy despite a strong correlation between inadequate literacy skills and worse disease knowledge. Although small sample size (352 patients with hypertension, 55 with diabetes) may have been a factor in that study, our results are similar in a much larger population.

In a study that examined the literacy level of children with type 1 diabetes and the literacy level of their mothers, Ross *et al*. reported no significant correlation between the mean annual A1C of the child and the child's literacy level[20]. However, a significant relationship was noted between the mean A1C of the child and the literacy level of the mother (*r *= 0.28; *P *= 0.01) after adjustment for the child's age and sex, duration of diabetes, daily insulin dose, the child's literacy score, and social class[20].

Similarly, Schillinger *et al*.[16] found that among 408 patients with type 2 diabetes seen in public hospital clinics, inadequate health literacy as measured by the S-TOFHLA, was independently associated with poor glycemic control (OR = 0.57; 95% CI 0.32–1.00; *P *= 0.05) retinopathy (OR = 2.33; 95% CI, 1.19–4.57; *P *= 0.01) and cerebrovascular disease (OR = 2.71; 95% CI, 1.06–6.97; *P *= 0.04). They controlled for age, sex, race/ethnicity, education, language, insurance, social support, depressive symptoms, complications of diabetes, diabetes education, duration of diabetes, and diabetes treatment. One explanation for our conflicting results may be related to differences in the populations under study. Schillinger's study involved a younger, more ethnically diverse population with poor glycemic control (mean A1C of 8.5%). Eighty-five percent of their population reported a race/ethnicity other than white, 46% did not complete high school, 32% had no health insurance, and 93% reported an annual household income less than $20,000. Another explanation for the difference between our outcomes and those reported by Schillinger *et al*. may be that our measurements were not exactly the same in all domains. We used different measures to assess depression and social support and different criteria for determining the presence of complications associated with diabetes.

Rothman *et al*. report that literacy was a statistically significant effect modifier in determining how patients with diabetes did in a disease management program; the patients with lower literacy were more likely to achieve goal glycemic control[21,22]. Consistent with our pre-intervention patient characteristics Rothman *et al*. reported similar baseline A1Cs between low and high literacy patients. However, in their study participation in a disease management program showed that literacy does appear to be a factor in diabetes control.

Our finding of a lack of significant association between literacy and disease outcome may be because compensatory strategies may have already been implemented to accommodate literacy barriers. It may be that achievement of some goals is more dependent on practitioner action than patient self-care skills[22]. Compared to the self-care associated with diabetes, the patient's role in management of cholesterol and blood pressure does not require as much complex reasoning and management. The older adults in the VDIS study may also have had support systems in place (help of a spouse, visiting nurse) to assist with diabetes management making health literacy less relevant. Medication tolerability may also be a factor for outcome achievement that goes beyond literacy.

Another possible explanation for our finding of a lack of a significant association between literacy and health outcomes is that optimal self-management of diabetes may not be solely dependent on reading ability. In addition to print literacy, health literacy includes numeracy, oral literacy, culture and context[2]. The published literature on "health literacy" typically reports measures of reading ability and rarely, if ever, addresses the broader domains of health communication[14,31,32]. While there may be great value in assessing health literacy to address communication barriers between patients and health care providers, we still do not know what aspects of health literacy may be important to communication, how to measure most aspects of health literacy, how to intervene, and if interventions will improve outcomes of care, particularly for adults with diabetes.

There are several additional limitations to our study. The cross-sectional study design does not allow us to measure incident outcomes or assign cause and effect. The generalizability of the sample is limited because it comes from a single region and is racially homogenous. There is a potential self-selection bias in the population of subjects who agreed to be interviewed, who differed significantly from the non-interviewed VDIS study population who were younger, more likely to be male, had better A1Cs and worse LDLs. The younger age group may have been more likely to be working and have other competing role demands limiting their availability to engage in an interview. It is also possible that subjects who declined participation in the interview process had lower literacy skills. However, all the subjects were outpatients with a diagnosis of diabetes and are representative of patients cared for in many community based primary care settings.

Most subjects had health insurance which can be accounted for by noting that the patients were recruited from primary care practices where they were receiving care, half were Medicare eligible, and Vermont has a low proportion of uninsured. The study population had relatively good glycemic control with almost half having an A1C < 7 %. The impact of literacy may be greater in a population with poorer glycemic control. Cognition is likely related to literacy and we did not specifically assess cognitive status but relied on exclusion criteria for the VDIS study which eliminated people with significant cognitive impairment. Data about diabetes complications were obtained by self-report and not verified by chart review. This may have resulted in some inaccuracies depending on a person's interpretation of the phrases chosen to query the presence of complications such as "problems with vision related to diabetes." Lastly, we note that multiple testing was performed; correction for this would have resulted in larger *P *values and not changed any of the conclusions.

## Conclusion

Our study suggests that literacy, as measured by the S-TOFHLA, is not associated with A1C, LDL, or BP in a cross-sectional study of older adults with diabetes under relatively good glycemic control. The common complications of diabetes are not associated with limited literacy in this population. Additional studies to examine the optimal measurement of health literacy and its relationship to health outcomes are needed.

## Competing interests

The author(s) declare that they have no competing interests.

## Authors' contributions

NSM made substantial contributions to analysis and interpretation of data, provided important intellectual content, and was involved in drafting and revising the manuscript. CDM made substantial contributions to conception and design; acquisition, analysis and interpretation of data; provided intellectual content and contributed to critical revision of the manuscript. BL made substantial contributions to conception and design; acquisition, analysis and interpretation of data; provided intellectual content and contributed to critical revision of the manuscript. All authors read and approved the final version of the manuscript.

## Pre-publication history

The pre-publication history for this paper can be accessed here:


